# Chronic red cell exchange in sickle cell patients with iron overload may not affect mortality

**DOI:** 10.3389/fmed.2022.892967

**Published:** 2022-09-27

**Authors:** Yi Yuan Zhou, Hollie M. Reeves, LaRhonda Webb, Zamira Santiago, Robert W. Maitta

**Affiliations:** ^1^University Hospitals Cleveland Medical Center, Cleveland, OH, United States; ^2^Case Western Reserve University School of Medicine, Cleveland, OH, United States

**Keywords:** sickle cell, iron overload, survival, red cell exchange, mortality

## Introduction

Sickle cell disease (SCD) is an inherited blood disorder that affects ~100,000 Americans (1, 2). In SCD, red blood cells (RBC) containing aberrant sickle hemoglobin (HgbS) become sickle-shaped at low oxygen tension and stick together leading to obstructed blood flow. The resulting decrease in tissue oxygenation causes chronic complications such as vasculo-occlusive pain crises among others that require frequent hospitalizations (3). Chronic RBC transfusions play a prominent role in the treatment of this disease by improving oxygenation through addition of normal RBC and dilution of RBC containing HgbS (4, 5). However, the major disadvantage of management with simple transfusion is the introduction of excess iron into the body which outpaces dedicated mechanisms for iron removal. Thus, over the course of the disease, excess iron is deposited into multiple organs causing organ damage leading to eventual organ failure. By contrast, automated red cell exchange (RCE) is an alternative therapeutic approach that exchanges the patient's sickle RBC with normal RBC reducing the sickle cell RBC more efficiently while being iron neutral (6). Despite the apparent advantage of RCE (7) and inclusion in current management recommendations (8), its benefits for patients with iron overload remains controversial. In this study we examined if RCE improved long-term survival for SCD patients with iron overload treated at our institution.

## Methods

The records of patients enrolled in the chronic RCE program at our institution, a tertiary academic medical center, were reviewed to identify patients treated for iron overload over a 2-year period. For each identified patient, multiple parameters were noted and followed including number of RCEs, procedure frequency, program adherence, pre- and post-procedural hematocrit and HgbS, serum ferritin, RBC units used in the exchange, and length of enrollment within the program. Study was approved by our institutional review board.

## Results

A total of 6 patients of 11 treated with chronic RCE for iron overload expired during the study period (Table 1). Patients were 3 females and 3 males with mean age of 44.2 years (range 30–67). Females were older at time of death (mean 56.3 years) compared to males (mean 32.0 years). An analysis of patients who expired showed that their pre-RCE HgbS were an average of 9.5% (range 2–20%). Patients received between 4 and 7 RBC units every 4–5 weeks with a target HCT of 28%, average target HgbS of 5.2% (range 1–10%). Overall patients who expired had poor tolerance to iron chelation. Serum ferritin measured toward the end of patients' lives was high (mean 5092.7 μg/L). Functional studies including iron-related imaging were not performed in the patients. The 6 patients who expired participated in the chronic RCE program for a mean duration of 13.2 months (range 2–21 months) and were compliant with scheduled exchanges. Patients died of cardiac complications, multiorgan failure, end stage renal and liver disease despite treatment adherence and maintenance of low HgbS concentration.

**Table 1:** Demographics and RCE parameters of study cohort.

**Age**	**Gender**	**Ht/wt (cm/Kg)**	**Pre-Hct (%)**	**Target Hct (%)**	**Pre-HgbS (%)**	**Target HgbS (%)**	**Ferritin (8-150 μg/L)**	**Units/RCE**	**Frequency**	**Duration in program**
62	F	162.6/56.2	20 (18–22)	28	15 (14–20)	10 (8–10)	5800	4	4 wks.	14 months
67	F	160/50.3	26 (23–33)	28	2 (1–2)	1	5580	4–5	4 wks.	2 months
30	M	190.5/110.7	23 (19–25)	28	10 (9–13)	5 (3–5)	3435	6–7	4–5 wks.	16 months
40	F	162.6/67.6	30 (26–33)	28	20 (19–22)	10 (9–15)	2941	5	4 wks.	7 months
35	M	162.6/58.1	20 (16–22)	28	5 (5–7)	3 (1–4)	8060	4	4 wks.	19 months
31	M	180.0/71.0	21 (20–22)	28	5 (4–8)	2 (2–4)	4740	4–5	4 wks.	21 months
44.2 56.3:32.0	3:3	169.2/69.0	23.3	28	9.5	5.2	5092.7	5	4	13.2 months

Hct, hematocrit; wt, weight; HgbS, hemoglobin S; pre-HgbS, mean and range is provided; RCE, automated red cell exchange. Target Hct and HgbS represent those expected at completion of RCE. Results represent mean/patient with range provided in parenthesis. Ferritin measurements are provided with normal range on top. For patients who had autopsies findings were consistent with end-stage renal disease, liver hemosiderosis, encephalopathy, myocardial infarction, diffuse intravascular coagulation and acute respiratory distress syndrome.

## Discussion

In our chronic RCE program, over half of patients treated for iron overload expired secondary to long-standing complications. Previous studies found that sudden death due to cardiac failure is the most common adverse event of chronic transfusions and results from chronic deposition of excess iron within the heart (9). Our data concur with these findings as all of the six expired patients died of cardiac complications with underlying multiorgan failure. Additionally, all 6 of the expired patients demonstrated chronic low pre-RCE HgbS concentration suggesting some degree of impaired RBC synthesis. Although conditions of excess iron such as hemochromatosis have been noted as a risk factor for anemia, the exact relationship between the two is still not completely understood (10). Furthermore, even though iron overload most commonly affect liver, heart, and endocrine systems, damage to organs related to hematopoiesis such as the kidneys can also occur (11). Regardless of the etiology for the low pre-RCE HgbS levels, the benefits of RCE in these patients is primarily improving patients' anemia while remaining iron neutral since HgbS at baseline was low. Finally, since RCE does not help actively remove excess iron, our results support reports that indicate that once iron is deposited in tissues the damage is often irreversible (12).

In the literature, data looking at adults treated via automated RCE for iron overload is mostly lacking. We present our experience which suggests a need for much larger studies with greater patients numbers to determine the benefit that RCE has in these patients. It would appear that stronger preventative measures, and earlier intervention against iron overload are more important to improve long-term survival of SCD patients. Although RCE can help slow iron overload-induced organ damage and prevent further iron deposition (8, 13), our data suggests that once sufficient organ damage has occurred, RCE may not have as much potency in improving patients' survival.

## Author contributions

YZ and HR gathered data and co-wrote the manuscript. LW and ZS gathered data. RM conceived study, gathered data, and edited manuscript to its final version. All authors contributed to the article and approved the submitted version.

## Conflict of interest

The authors declare that the research was conducted in the absence of any commercial or financial relationships that could be construed as a potential conflict of interest.

## Publisher's note

All claims expressed in this article are solely those of the authors and do not necessarily represent those of their affiliated organizations, or those of the publisher, the editors and the reviewers. Any product that may be evaluated in this article, or claim that may be made by its manufacturer, is not guaranteed or endorsed by the publisher.

## References

[1] Zheng Y Maitta RW. Alloimmunisation rates of sickle cell disease patients in the United States differ from those in other geographical regions. Transfus Med. (2016) 26:225–30. 10.1111/tme.12314 27197689

[2] Brousseau DC Panepinto JA Nimmer M Hoffmann RG. The number of people with sickle-cell disease in the United States: national and state estimates. Am J Hematol. (2010) 85:77–8. 10.1002/ajh.21570 20029951

[3] Kauf TL Coates TD Huazhi L Mody-Patel N Hartzema AG. The cost of health care for children and adults with sickle cell disease. Am J Hematol. (2009) 84:323–7. 10.1002/ajh.21408 19358302

[4] Yawn BP Buchanan GR Afenyi-Annan AN Ballas SK Hassell KL James AH. Management of sickle cell disease: summary of the 2014 evidence-based report by expert panel members. JAMA. (2014) 312:1033–48. 10.1001/jama.2014.10517 25203083

[5] Yazdanbakhsh K Ware RE Noizat-Pirenne F. Red blood cell alloimmunization in sickle cell disease: pathophysiology, risk factors, and transfusion management. Blood. (2012) 120:528–37. 10.1182/blood-2011-11-327361 22563085 PMC3401213

[6] Maitta RW. Current state of apheresis technology and its applications. Transfus Apher Sci. (2018) 57:606–13. 10.1016/j.transci.2018.09.009 30220449

[7] Fasano RM Leong T Kaushal M Sagiv E Luban NL Meier ER. Effectiveness of red blood cell exchange, partial manual exchange, and simple transfusion concurrently with iron chelation therapy in reducing iron overload in chronically transfused sickle cell anemia patients. Transfusion. (2016) 56:1707–15. 10.1111/trf.13558 26997031

[8] Chou ST Alsawas M Fasano RM Field JJ Hendrickson JE Howard J. American Society of Hematology 2020 guidelines for sickle cell disease: transfusion support. Blood Adv. (2020) 4:327–55. 10.1182/bloodadvances.2019001143 31985807 PMC6988392

[9] Kohgo Y Ikuta K Ohtake T Torimoto Y Kato J. Body iron metabolism and pathophysiology of iron overload. Int J Hematol. (2008) 88:7–15. 10.1007/s12185-008-0120-5 18594779 PMC2516548

[10] Khan AA Hadi Y Hassan A Kupec J. Polycythemia and anemia in hereditary hemochromatosis. Cureus. (2020) 12:e7607. 10.7759/cureus.7607 32399341 PMC7213665

[11] McLaren GD Muir WA Kellermeyer RW. Iron overload disorders: natural history, pathogenesis, diagnosis, and therapy. Crit Rev Clin Lab Sci. (1983) 19:205–66. 10.3109/10408368309165764 6373141

[12] van Tuijn CF Schimmel M van Beers EJ Nur E Biemond BJ. Prospective evaluation of chronic organ damage in adult sickle cell patients: a seven-year follow-up study. Am J Hematol. (2017) 92:E584–E90. 10.1002/ajh.24855 28699283

[13] Tsitsikas DA Sirigireddy B Nzouakou R Calvey A Quinn J Collins J. Safety, tolerability, and outcomes of regular automated red cell exchange transfusion in the management of sickle cell disease. J Clin Apher. (2016) 31:545–50. 10.1002/jca.21447 26878828

